# Canopy Venom: Proteomic Comparison among New World Arboreal Pit-Viper Venoms

**DOI:** 10.3390/toxins8070210

**Published:** 2016-07-08

**Authors:** Jordan Debono, Chip Cochran, Sanjaya Kuruppu, Amanda Nouwens, Niwanthi W. Rajapakse, Minami Kawasaki, Kelly Wood, James Dobson, Kate Baumann, Mahdokht Jouiaei, Timothy N. W. Jackson, Ivan Koludarov, Dolyce Low, Syed A. Ali, A. Ian Smith, Andrew Barnes, Bryan G. Fry

**Affiliations:** 1 Venom Evolution Lab, School of Biological Sciences, University of Queensland, St Lucia, QLD 4072, Australia; jordan.debono@uqconnect.edu.au (J.D.); gone2@westnet.com.au (K.W.); james.dobson@uqconnect.edu.au (J.D.); kate.baumann@uqconnect.edu.au (K.B.); mahdokht.jouiaei@gmail.com (M.J.); tnwjackson@gmail.com (T.N.W.J.); jcoludar@gmail.com (I.K.); dol_yce@hotmail.com (D.L.); dr.syedabidali@gmail.com (S.A.A.); 2 Department of Earth and Biological Sciences, Loma Linda University, Loma Linda, CA 92350, USA; rcochran@llu.edu; 3 Department of Biochemistry & Molecular Biology, Biomedical Discovery Institute, Monash University, Clayton, VIC 3800, Australia; sanjaya.kuruppu@monash.edu (S.K.); Ian.Smith@monash.edu (A.I.S.); 4 School of Chemistry and Molecular Biosciences, University of Queensland, St Lucia, QLD 4072, Australia; a.nouwens@uq.edu.au; 5 Baker IDI Heart and Diabetes Institute, 75 Commercial Road, Melbourne, Victoria 3004, Australia; niwanthi.rajapakse@bakeridi.edu.au; 6 Department of Physiology, Biomedical Discovery Institute, Monash University, Clayton, VIC 3800, Australia; 7 Aquatic Animal Health, School of Biological Sciences, University of Queensland, St Lucia, QLD 4072 Australia; minami.kawasaki@uqconnect.edu.au (M.K.); a.barnes@uq.edu.au (A.B.); 8 Institute for Molecular Bioscience, University of Queensland, St Lucia, QLD 4072, Australia; 9 HEJ Research Institute of Chemistry, ICCBS, University of Karachi, Karachi-75270, Pakistan

**Keywords:** venom, evolution, pit-viper, enzyme

## Abstract

Central and South American pitvipers, belonging to the genera *Bothrops* and *Bothriechis*, have independently evolved arboreal tendencies. Little is known regarding the composition and activity of their venoms. In order to close this knowledge gap, venom proteomics and toxin activity of species of *Bothriechis*, and *Bothrops* (including *Bothriopsis*) were investigated through established analytical methods. A combination of proteomics and bioactivity techniques was used to demonstrate a similar diversification of venom composition between large and small species within *Bothriechis* and *Bothriopsis*. Increasing our understanding of the evolution of complex venom cocktails may facilitate future biodiscoveries.

## 1. Introduction

Snake venoms are complex secretions of various enzymes, peptides and small organic molecules that have diverse modes of action on both prey and accidental human bite victims [1,2,3,4,5,6]. Though targeting a wide array of physiological processes and targets, the proteins that make up snake venom come from relatively few protein families. This is due to core protein scaffolds being maintained with accelerated rates of evolution at the secondary through quaternary levels facilitating the evolution of multiple derived functions within each protein family [3,7,8,9]. The use of mass spectrometry in venom proteomics [7,10,11,12,13,14,15,16,17,18,19] and venom gland transcriptome analysis [9,20,21,22,23,24,25,26,27,28] has improved knowledge of venom composition tremendously. Variation in venom profiles has been shown interspecifically [7,13,18,19,29,30,31,32,33,34] and intraspecifically, with intraspecific differences found among geographic locales [12,35,36,37,38,39,40,41,42,43], between sexes [36,37,44,45], and between age classes [10,15,46,47,48,49,50,51,52]. Some authors have argued that diversity in venom composition is the result of neutral evolutionary processes and is not subject to natural selection [53,54], whereas others have argued that adaptation has been driven, by strong natural selection, to specific prey species [19,29,36,37,55,56,57,58,59,60].

**Figure 1:**
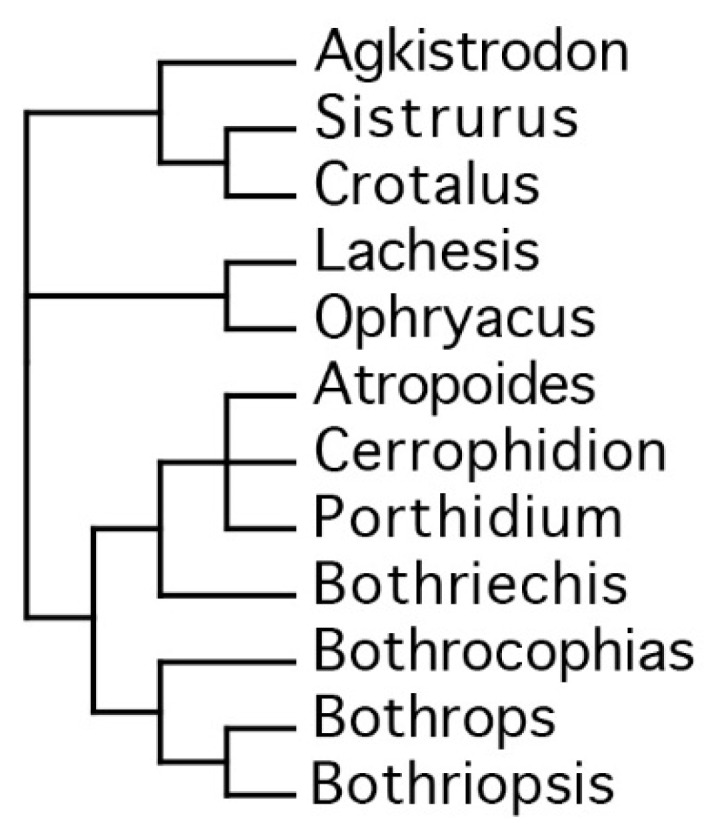
Taxonomical relationships of American pitvipers, based on Castoe 2006 [65].

New World pitvipers are believed to be descendants of an ancestral Asian pitviper species, by way of a single New World colonization event, via the Bering land bridge [61,62,63,64]. In regards to the current study particular interest is given to the members of the genera *Bothriechis* and *Bothrops* that have independently evolved to occupy arboreal niches. That *Bothriechis* is a distinctive genus from the “bothropoid” genera [61] has been asserted by multiple authors [61,62,63,64,65,66,67]; however, phylogenetic relationships within the bothropoids, in particular the validity of *Bothriopsis,* have proven more difficult due to limited sampling of taxa. Numerous studies have presented evidence of the paraphyly of *Bothrops* with respect to *Bothriopsis* [61,62,63,64,65,66,67,68,69,70,71,72] leading some authors to advocate for the synonymizing of *Bothriopsis* and *Bothrocophias* with *Bothrops* [70,72] or *Bothriechis* [73], while others have maintained that *Bothriopsis* and *Bothrocophias* should remain distinct [61,62,65,74,75] (Figure 1). Regardless of taxonomical divisions preferred, it is clear that the arboreal specialization has occurred upon two separate occasions.

Recent studies utilizing a combined morphological/molecular approach and increased sampling of taxa have disagreed on generic assignment as well. Fenwick *et al.* (2009), sampling 90% of the known “bothropoid” taxa and utilizing both morphological characters and mtDNA sequence data found support for several monophyletic clades within *Bothrops* (*sensu lato*) and proposed taxonomic revisions, including recognition of *Bothriopsis*, based on the distinct species groups recovered [76]. Carrasco *et al.* (2012) [77] incorporated additional morphological characters not utilized by Fenwick *et al.* (2009) [76] in addition to previously published molecular sequences and ecological characters. Citing possible paraphyly of *Bothrops* with respect to *Bothriopsis* (with the inclusion of *Bothrops sanctaecrucis*)*,* they proposed following the recommendation of Salomão *et al.* (1997) [70] and Wüster *et al.* (2002) [72] and synonymized *Bothriopsis* with *Bothrops* [77]. We have followed the proposals of Salomão *et al.* (1997) [70] and Wüster *et al.* (2002) [72] in recognizing the bothropoids as a single genus, *Bothrops*, as the weight of current evidence (cited above) favors this classification.

Despite the medical significance of pitviper envenomations in the New World [78,79,80,81,82,83], the majority of toxinological research published to date has been on North American members of the genus *Crotalus* [14,25,26,35,38,39,40,41,42,43,49,50,82,83,84,85,86,87,88,89,90], and the larger members of the genus *Bothrops* [10,91,92,93,94,95,96,97,98,99,100,101,102,103,104]. While investigations into *Bothriechis*, in particular *B*. *schlegelii*, venom [31,105,106,107] and some of the smaller bothropoid pitvipers [108,109,110,111] have occurred, knowledge gaps remain on the proteomic make up of the venom of the majority of New World pitviper species. This knowledge gap is unfortunate as snake venoms have proved valuable sources of investigational ligands [112,113,114,115,116] and potential therapeutics [117,118,119,120,121,122,123,124] due to the high degree of target specificity exhibited by their constituent toxins. *Bothrops and Bothriechis* are both medically relevant species across their range and together are responsible for a large number of severe envenomations [78,79,80,81,125,126]. American pitviper venoms are typically rich in two types of enzymes: Snake venom metalloprotease (SVMP) and phospholipase A_2_ (PLA_2_) [3,42,127]. Due to variance in retention of ancestral domains, SVMPs span a broad range of molecular weights from ~30–70 kDa and exhibit a variety of activities. Structural diversity within SVMPs is the basis for the development of a variety of symptoms in victims of envenomation including; haemorrhage, edema, hypotension, hypovolemia, inflammation, and necrosis [128,129]. The realities of inter/intraspecific venom variation necessitate a thorough understanding of species venom proteomes as the information allows for improved clinical treatment of envenomations by way of informing antivenom selection for heterologous venoms [127,130]. Determining a species proteome is often the first step of antivenomic studies undertaken to confirm antivenom neutralization efficacy of species/populations/age classes [12,84,131] and determination of toxins unable to be neutralized is valuable information not only for clinicians in charge of managing envenomations but also for future antivenom production.

In this study we investigate the venom proteomes of arboreal members of the *Bothriechis* and *Bothrops* genera that have independently invaded the tree canopies of South America [7,31,64,71,77,107]. In both genera variation in adult size has been documented, with *Bothriechis aurifer, Bothriechis lateralis* and *Bothriechis marchi* typically exceeding *Bothriechis schlegelli* in adult size, while *Bothrops bilineata* is a much smaller species than *Bothrops taeniata*. For comparison, we have included two terrestrial *Bothrops* species that also differ markedly in maximum adult size, with *Bothrops asper* reaching adult sizes in excess of twice that of *Bothrops neuwiedi bolivianus*. While *B. lateralis* and *B. schlegelli* have been proteomically previously profiled, as has *Bothriechis nigroviridis* [31,132], their venom variation has not been considered in relation to size and prey preference.

## 2. Results

Shotgun mass spectrometry recovered proteins of known pitviper toxin types (Table 1) in agreement with previous proteomic [10,11,13,14,30,32,83,84,92] and transcriptomic analyses [22,24,29,59,77,89,90,93,95,97,103].

**Figure 2:**
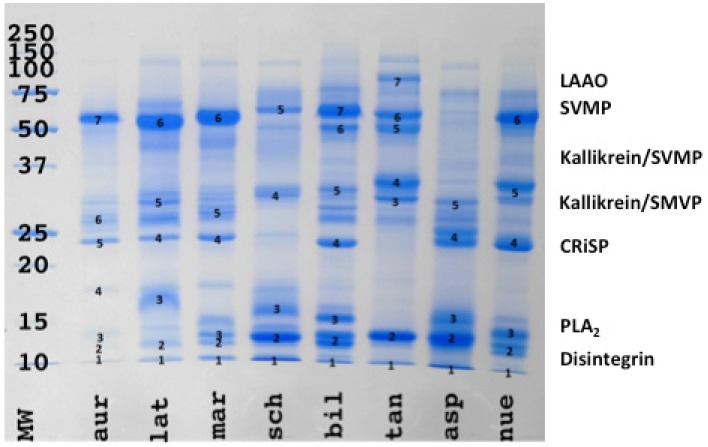
1D SDS page. MW = molecular weight marker; aur = *Bothriechis aurifer*; lat = *Bothriechis lateralis*; mar = *Bothriechis marchi*; sch = *Bothriechis schlegelli*; bil = *Bothrops bilineata*; tan = *Bothrops taeniata*; asp = *Bothrops asper*; neu = *Bothrops neuwiedi bolivianus*. Annotation indicates the dominant type in a region. However, other toxin types may also be present (see Supplementary File 1 for full annotation).

**Figure 3:**
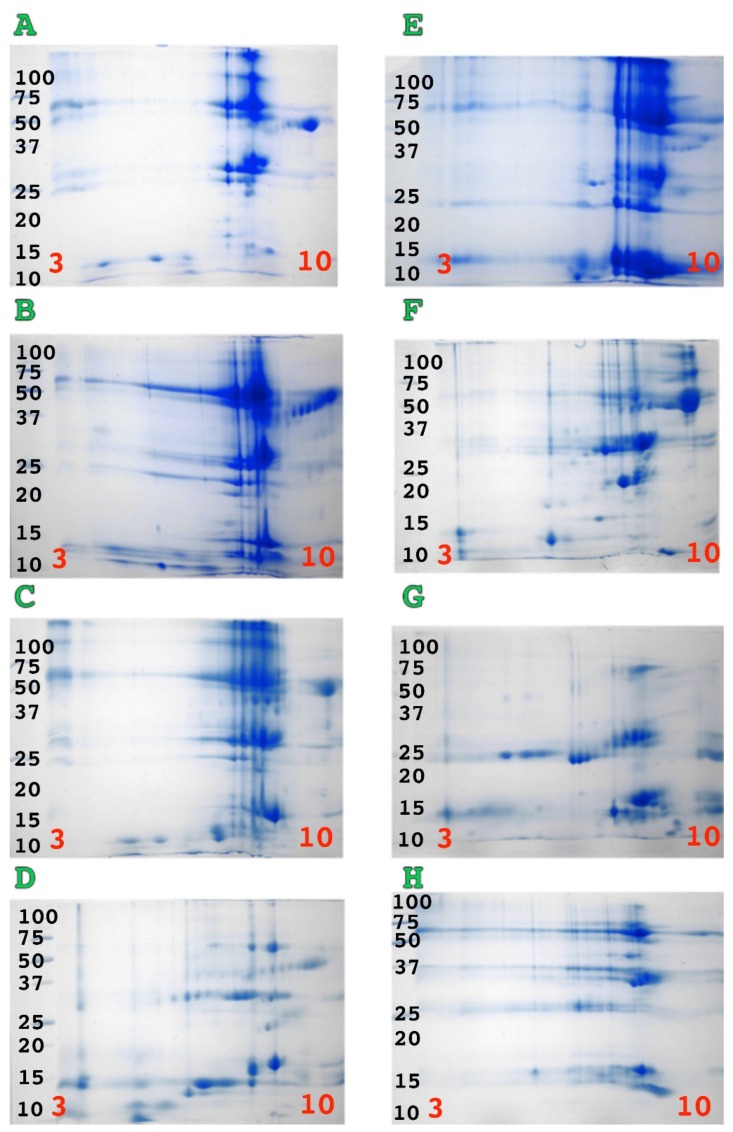
2D SDS page analysis of (**A**) *Bothriechis aurifer*; (**B**) *Bothriechis lateralis*; (**C**) *Bothriechis marchi*; (**D**) *Bothriechis schlegelli*; (**E**) *Bothrops bilineata*; (**F**) *Bothrops taeniata*; (**G**) *Bothrops asper* and (**H**) *Bothrops neuwiedi bolivianus*. pI range is 3–10 (left to right) and molecular weight markers are as for Figure 2.

1D gel analysis revealed greater complexity in all venoms than indicated by the shotgun results. The venoms of the larger *Bothriechis* species (*B. aurifer, B. lateralis* and *B. marchi*) contained more P-III, P-II and P-I SVMP than the venom of the smaller sized species *B. schlegelii* (Figure 2). In contrast, *B. schlegelii* venom contained more PLA_2_ (lower molecular weights of synovial PLA_2_ 12–15 kDa). A similar correlation between body size and SVMP/PLA_2_ content was found in arboreal *Bothrops*. However this trend was reversed in the terrestrial *Bothrops*. These results were complemented by the 2D gel analyses (Figure 3).

**Figure 4:**
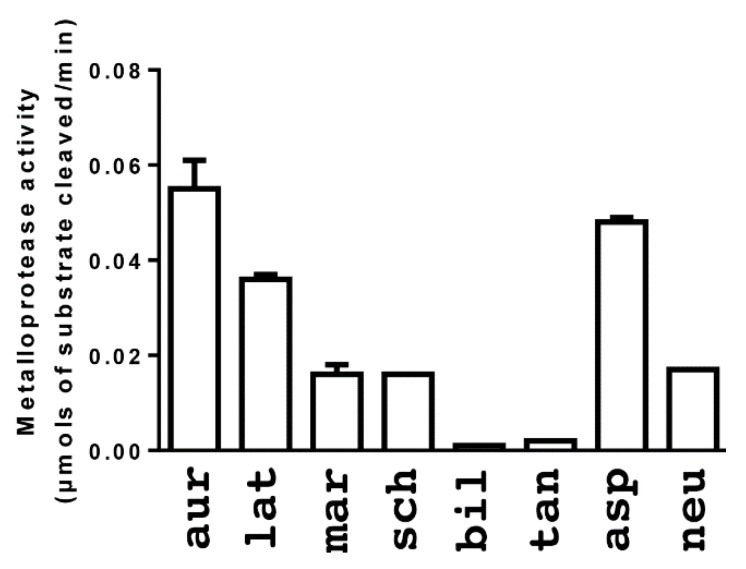
Metalloprotease activity of venom—Metalloprotease activity of venom (10 ng/µL) was measured based on its ability to cleave a fluorogenic peptide substrate (Mca-PLGL-Dpa-AR-NH_2_, 10 µM final). aur = *Bothriechis aurifer;* lat = *Bothriechis lateralis;* mar = *Bothriechis marchi;* sch = *Bothriechis schlegelii*; bil = *Bothrops bilineata;* tan = *Bothrops taeniata;* asp = *Bothrops asper;* neu = *Bothrops neuwiedi bolivianus.*

**Figure 5:**
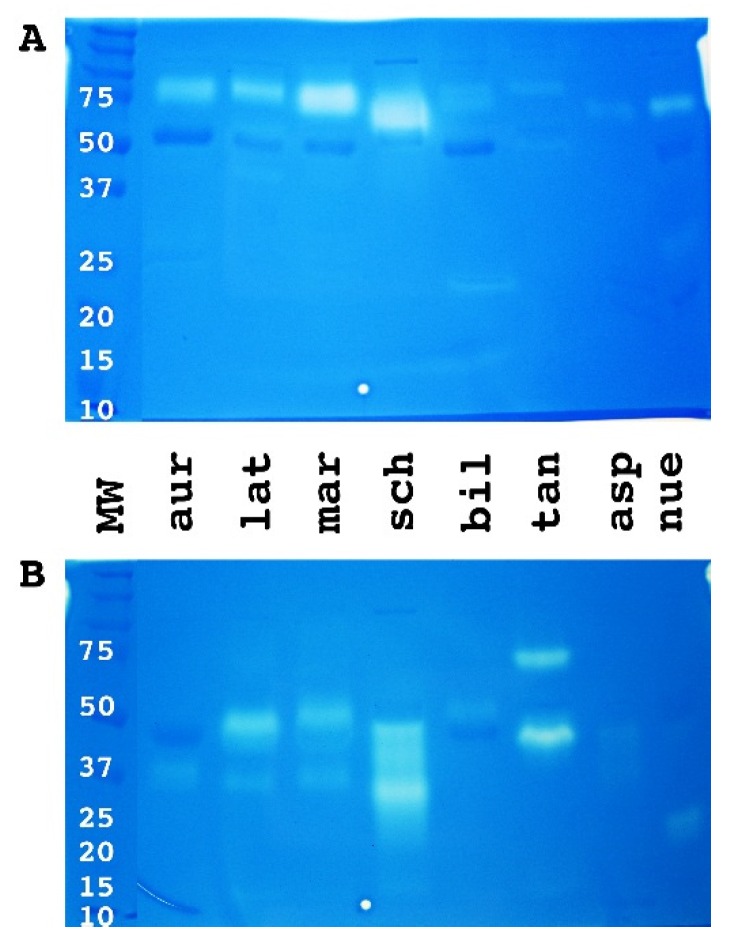
Crude venom zymography gel analysis using casein (**A**) and gelatin (**B**) as protein substrates. MW = molecular weight marker; aur = *Bothriechis aurifer;* lat = *Bothriechis lateralis;* mar = *Bothriechis marchi;* sch = *Bothriechis schlegelii*; bil = *Bothrops bilineata;* tan = *Bothrops taeniata;* asp = *Bothrops asper;* neu = *Bothrops neuwiedi bolivianus.*

The relative straightforward proteomics trends were not reflected by the most complex differences in venom composition revealed by the bioactivity testing, which is consistent with venoms being complex mixtures of bioactive substances. While *B. aurifer* was indeed much more potent than *B. schlegelli* in a fluorescence-based metalloprotease activity assay (Figure 4), and *B. lateralis* also more active, *B. marchi* was only weakly active, being equipotent to *B. schlegelli.* This result was despite *B. marchi* having very similar 1D and 2D gel patterns to the more potent *B. lateralis,* especially in the known metalloprotease regions. It must be noted that these trends are of course specific only to this particular substrate and that other assays (such as the gel based zymography) may present differential results. Indeed, further investigation of metalloprotease activity yielded results that contrasted those gathered with the aforementioned analyses (Figure 4). Zymography gels with a casein substrate revealed active P-III SVMP in all four *Bothriechis* venoms in the 75 kDa region (Figure 2). The region of digestion in the casein zymography gel (Figure 5) corresponds not with the 50 kDa heavy bands seen in the 1D gels (Figure 2), but a 75 kDa region above that, with the 50 kDa 1D bands clearly evident in the zymography gels below the zone of digestion. This suggests that this digestive activity is due to the presence of heavily glycosylated enzymes that are resistant to staining, while the well-stained 50 kDa serine protease bands do not have this type of activity. In contrast, zymography gels with gelatin as the substrate produced bands of digestion in the *Bothriechis* venoms at the 50 kDa region (Figure 5), except for *B. aurifer* despite it having seemingly homologous serine protease band in the same region.

The arboreal *Bothrops* venoms were both weakly active on the casein substrate zymography gel (Figure 5). However, these two venoms differed sharply in the gelatin substrate zymography gel, with *B. taeniata* having two discrete zones of digestion, one of which was similar to that of the *Bothriechis* species, while the other was higher in molecular weight (Figure 5). Neither arboreal *Bothrops* venom, however, displayed significant activity in the fluorescence based assay. Both terrestrial *Bothrops* species were only weakly active in either the casein or gelatin (Figure 5) substrate zymography gels. However both were active in the fluorescence based metalloprotease assay, with *B. asper* displaying high levels of activity.

**Figure 6:**
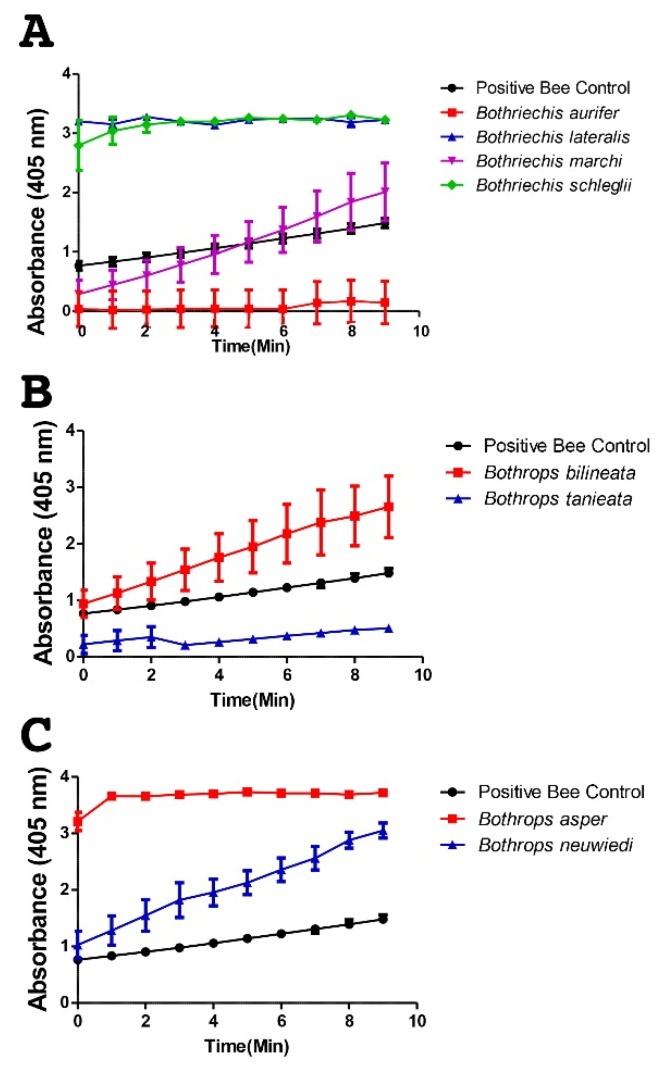
Phospholipase A_2_ enzymatic activity profiling measured by means of absorbance over time at 1 µg for (**A**) *Bothriechis* species; (**B**) arboreal *Bothrops* species and (**C**) terrestrial *Bothrops* species.

PLA_2_ activity levels, however, were more congruent with the relative presence of venom components in the corresponding range (12–15 kDa), with *B. aurifer* and *B. marchi* having only weak activity while *B. lateralis* and *B. schlegelli* were very active (Figure 6). This is consistent with the latter two species having darker staining bands at ~12–15 kDa than the former two species. Thus envenomations by *B. lateralis* and *B. schlegelli* would be expected to produce more myotoxicity than envenomations by *B. aurifer* and *B. marchi*. Despite *Bothrops bilineata* and *B*. *taeniata* both containing abundant PLA_2_, they were only weakly active in this region, suggesting that myotoxicity would not be as significant a complication but that other non-enzymatic PLA_2_ activities (e.g., antiplatelet aggregation or neurotoxicity) may be more potent in these two species. Similarly, despite both being rich in PLA_2_, the two terrestrial *Bothrops* species displayed significantly different levels of PLA_2_ enzymatic activity. *Bothrops asper* displayed high levels of PLA_2_ enzymatic activity, consistent with myotoxic envenomation effects, while *Bothrops neuwiedi bolivianus* was much less potent in this regard.

## 3. Discussion

For the *Bothrops* species tested, SVMP levels are correlated with consumed prey class percentages, reported by Martins et al. (2002) [132], with species containing higher percentage of anurans in their diet (*B*. *bilineata* and *B*. *taeniata*) possessing lower levels of SVMP activity than species that contained non-volant mammals as a higher percentage of their diet (*B*. *asper* and *B*. *neuwiendi*). Pitviper venoms have been dichotomously classified into type 1 (proteolytic or “tenderizer”) and type II (more toxic) venoms [133], as anurans have a high surface area to volume ratio and skin lacking tough dermal scales, it is unlikely that strong selection pressures for venoms with high metalloprotease activity exist in species that contain anurans as a large percentage of their diet as the need for such cleavage enzymes is reduced. The increased levels of SVMP activity for non-volant mammal generalist *B*. *asper* (59.4% mammals) in regards to *B*. *neuwiedi* (93.1% mammals) may be due to size class of mammal taken, as pitvipers are gape-limited predators that consume their prey whole [134,135,136]. Pitviper SVL (snout vent length) in the North American pitviper, *Crotalus atrox*, was found as the best predictor of overall maximum gape [137]. Although *B. asper* is recorded as consuming less percentage of mammals than *B. neuwiedi*, *B*. *asper* obtains an average SVL length roughly twice that of *B*. *neuwiedi* [131], suggesting the requirement for increased amounts of SVMP, as larger prey requires increased levels of SVMP which impairs homeostasis during predation. In addition our venom samples were taken from adult individuals displaying the predicted adult requirement of toxin activity. Other studies have documented that pitvipers consume prey species with lower surface area to volume ratios as they grow [50,132,138,139] and the lytic activities of SVMP may aid in predigestion of acquired prey. A detailed dietary analysis including prey species/size would be necessary in order to determine if *B*. *asper* does indeed consume larger prey/ prey of lower surface area to volume ratio than *B*. *neuwiedi*.

Detailed dietary studies for members of *Bothriechis* are lacking. As a whole the genus appears to be a generalist with an ontogenetic shift toward mammalian prey, with *B*. *lateralis* and *B*. *schlegelii* even documented as occasionally predating on bats and small birds (Campbell and Lamar 2004 and references therein) [131]. We detected increased levels of SVMP activity for *B*. *aurifer* in relation to other members of *Bothriechis*. This increased level of SVMP activity in combination with Campbell and Lamar (2004) [131] noting that the snake is frequently encountered on the ground is suggestive that *B*. *aurifer* may contain a higher percentage of non-volant mammals in its diet than the other members of *Bothriechis* tested. In contrast to high activity levels of SVMP recorded in *B. aurifer*, *B. schleglii* and *B. lateralis* both present higher levels of sPLA_2_ activity in relation to other members of *Bothriechis*. The increased levels of sPLA_2_ activity present in the venom profiles of *B. lateralis* and *B. schleglii* is suggestive of their arboreal encounters with volant animals. As sPLA_2_ is a neurotoxin affecting the nervous system, recruiting such a toxin would aid in quickly immobilizing flying prey whilst ambushing from a tree branch high up in the forest canopy, therefore reducing the risk of escape and injury.

Detailed knowledge of venom components present in *Bothrops* and *Bothriechis* venoms has the ability to improve treatment for those receiving an envenomation [12,15,84,126,130,140,141]. This is an important task as the two genera are responsible for a considerable number of bites [78,79,80,81,82,83] and knowledge of inter and intraspecific venom variation is crucial for optimizing neutralization capabilities of antivenoms cf. [84,126].

Further research into the identification and activity of toxins present in the venoms of snakes within this clade will be conducted in order to elucidate the selection pressures that have shaped these fine-tuned weapon systems. This research will hopefully contribute towards filling existing knowledge gaps concerning the evolution of venom composition and activity in the Viperidae.

## 4. Experimental Section

### 4.1. Venom Collection

A series of 8 snake species were chosen due to their phylogenetic positioning, genetic relatedness, body size/length variation, and lack of existing knowledge of their venom composition for proteomic analyses. Pooled venom samples were collected from both from adult wild and captive specimens residing in private collections. Venom samples were collected by inducing the snake to bite a sterile container and collected venom was immediately stored on dry ice or in liquid nitrogen during transport. Once in the laboratory, samples were stored at −80 °C after lyophilization. Species investigated were *Bothriechis aurifer* (captive snakes of unknown locality), *Bothriechis lateralis* (captive snakes of unknown locality), *Bothriechis marchi* (captive snakes of unknown locality), *Bothriechis schlegelii* (Costa Rica), *Bothrops bilineata* (French Guyana), *Bothrops taeniata* (captive snakes of unknown locality), *Bothrops asper* (Costa Rica), and *Bothrops neuwiedi bolivianus* (Bolivia).

### 4.2. Proteomics

#### 4.2.1. 1D and 2D Gels Using SDS PAGE Electrophoresis Methodology

##### 1D SDS-PAGE

Reduced 1D Tris-glycine SDS-PAGE methodology followed that of the study conducted by Ali et al., 2013 (cf. [11]). Specific changes were as follows: samples (20 ug) loaded were 15 µL total volume, gels were run at 90 V for 20 mins and then 120 V for 40 min, and gels were de-stained in 100 mL Milli Q.

##### Mini 2D SDS-PAGE

###### First Dimension

A sample of 125 µL solubilization buffer was added to a pre-washed sample of 300 µg of protein from required venom stock, prepared in a 15 mL Falcon tube. A solution of 0.75 µL of Bio-Lyte Ampholyte was added to the buffer and gently mixed. A sterile IPG strip (Bio-Rad Ready Strip Oakland, California non-linear pH 3–10, 7 cm, Hercules, CA, USA) was then added to the Corning Falcon Conical tube (Tamaulipas, Mexico) and allowed to absorb the solution overnight.

IPG strips were removed from Falcon tubes and placed gel side up an Isoelectric Focusing (IEF) machine (PROTEAN i12 IEF CELL Bio-RadLab Oakland, Hercules, CA, USA). Strips were covered with mineral oil and run overnight. Running conditions followed that of Ali et al., 2013 (cf. [11]).

###### Second Dimension

Required mini gels (7 cm × 6 cm) were cast using the same protocol for 1D Tris-glycine SDS-PAGE resolving layers (without the stacking layer). A layer of gel-overlay buffer (1 mL) was placed on top of the resolving layer once dry. A solution of 38 mg dithiothreitol (DTT) and 2.5 mL Equilibration buffer was added to each strip and gently mixed for 10 min. DTT solution was removed and a solution of 50 mg iodoacetamide (IAA) and 2.5 mL Equilibration buffer, gently mixed for 20 min.

Gel-overlay buffer was removed and 1 mL liquid agarose was laid over the gel. IPG strips were then embedded into the agarose layer, positive end on the left. A molecular marker gel bit was then inserted on the negative end. Gels were then placed in the electrophoresis container with a combination of 10× electrode buffer and Milli Q (80:800 mL). Power pack was set at 20 mA for 60 min and run at room temperature. 

Once gels were finished they were removed from their glass cast and placed in 10 mL Coomassie brilliant blue stain, and set on a rocker overnight.

##### Liquid Chromatography-tandem Mass Spectrometry (LC-MS/MS) Analyses

In order to identify the types of toxins present in each gel, bands/spots from 1D SDS-PAGE glycine gels were picked, digested and analysed by LC-MS/MS. Visible protein bands were cut and stored at −80 °C. De-stain solution (500 µL 50 mM Ammonium bicarbonate (ABC)/50% Acetonitrile (ACN)) was added to each picked band/spot and left overnight. The destain was removed and repeated.

###### Reduction and Alkylation

Once blue stain had disappeared from picked bands, destain was removed. For 1D bands, a solution of 40 µL of 10 mM DTT was added to each band. Bands were then incubated at 60 °C for 30 min reducing disulphide bonds, and DTT removed. A solution of 40 µL of 55 mM IAA was added to the bands and incubated for 30 min at room temperature in the dark, alkylating free cysteine residues. Once IAA was removed, 100 µL of 50 mM ABC wash was added. Tubes were then spun down using a vortex for 2 min, ABC wash was removed and process repeated, washing away any excess IAA (removing IAA from the samples was important, as the solution can create adducts on proteins of interest). Remaining IAA was removed, and 100 µL of 100% ACN was added and left for minimum 5 min. ACN dehydrates the gel pieces, turning them white. Once gel pieces had turned white, ACN was removed.

###### Enzymatic Digestion

Gel pieces were rehydrated with 8 µL trypsin, brought up in hydrochloric acid (10 ng/µL, further diluted in 50mM ABC). This was left for 10–20 min at 4 °C. Depending on gel piece size, an additional 6 µL to 16 µL 50 mM ABC buffer was added. Gel pieces were kept moist overnight during digestion, incubated at 37 °C.

###### Peptide Extraction

Digested peptides were resuspended in 10 µL of 2.5% ACN/0.1% FA. Samples were filtered via ziptips using the following protocol; tips were wetted with 100% ACN (3 × 10 µL), and equilibrated with 10 µL 5% ACN/0.1% TFA, also repeated 3 times. Samples were loaded and pipetted up and down at least twice the volume of sample. Tip was washed with 5% ACN/0.1% TFA (3 × 10 µL). Samples were eluted with 80% ACN/0.1% TFA (10 µL) in a new tube. Combined samples were transferred into glass vials and diluted with 0.1% FA (final loading volume 20 µL).

For LC-MS/MS analysis, parameters are as follows; samples were separated using RP-chromatography on a Dionex Ultimate 3000 RSLC nano-system (Lifetech, Carlsbad, CA, USA). Samples were desalted on a Thermo PepMap 100 C18 trap (Lifetech, Carlsbad, CA, USA) (0.3 × 5 mm, 5 µm) for 5 min with a flow rate of 30 µL/min. This was followed by separation on an Acclaim PepMap RSLC C18 (Lifetech, Carlsbad, CA, USA) (150 mm × 75 µm) column at a flow rate of 300 nL/min. A gradient of 10%–70% buffer B over 7 min where buffer A (1% ACN/0.1% FA) and buffer B (80% ACN/0.1% FA) was used to separate peptides. Eluted peptides were directly analysed on an Orbitap Elite mass spectrometer (Thermo Scientific, Carlsbad, CA, USA) using an NSI electrospray interface. Source parameters included a capillary temperature of 275 °C; S-Lens RF level at 60%; source voltage of 2 kV and maximum injections times of 200 ms for MS and 150 ms for MS2. Instrument parameters included an FTMS scan across m/z range 350–1800 at 60,000 resolution followed by information dependent acquisition of the top 10 peptides across m/z 40-1800. Dynamic ion exclusion was employed using a 15 s interval. Charge state screening was enabed with rejection of +1 charged ions and monoisotopic precursor selection enabled. Data was converted to mascot generic format (mgf) using the msConvert software (ProteoWizard v2.0) and searched using Protein Pilot™ v5.0 (Sciex). 

#### 4.2.2. Shotgun Analysis

##### Reduction, Alkylation and Trypsinization

Pre washed 10 µg protein venom samples, solubilised with 40 µL of Milli Q, was added to 5 µL of 1 M Ammonium Carbonate. A total of 55 µL per sample (97.5% acetonitrile, 2% iododethanol and 0.5% ttriethylphosphene) was added, with a total combined volume of 100 µL. Samples were then incubated at 37 °C for 2 h, and then freeze-dried. Samples were re-suspended in 25 µL of 40 mM ammonium bicarbonate. Trypsin (1 ng) was added to each re-suspended sample and incubated at 37 °C overnight. Samples were dried and re-suspended in 40 µL 5% acetonitrile and 1% formic acid in preparation for LC-MS-MS analysis according to methods described in detail elsewhere [11].

### 4.3. Bioactivity Testing

#### 4.3.1. Fluorescent Determination of Metalloprotease Activity

Freeze dried venom was reconstituted in a buffer containing 150 mM NaCl and 50 mM Tri-HCl (pH 6.3). Metalloprotease activity in venom (10 ng/µL) was measured by adding quenched fluorescent substrate (10 µM final; Fluorogenic Peptide Substrate, R & D systems, Cat#ES001, Minneapolis, Minnesota). Fluorescence was monitored (excitation at 320 nm and emission at 405 nm) over 100 min, and rate of substrate cleavage calculated from a standard curve of known fluorophore (7-methoxycoumarin) concentrations. The specificity of metalloprotease activity was confirmed by incubating venom for 24 h at 37 °C with EDTA (50 mM).

#### 4.3.2. Zymogram PAGE 10% and 12% Gels Bio-Rad

Dried samples of 30 µg were prepared for each species and reconstituted in 15 µL of Bio-Rad Sample Buffer (#161-0764 supplied). Bio-Rad Zymogram PAGE ready-made 10% gelatin and 12% casein gels (1,611,113 and 1,611,114 respectively) were run using standard PAGE protocol at 100V for 90 min in 1× Tris-Glycine SDS Running Buffer (working concentration 25 mM Tris, 192 mM glycine, 0.1% SDS). Gel was removed from case and incubated at room temperature for 30 min in 100 mL 1× Zymogram Renaturing Buffer (#161-0765, 2.5% Triton ×100, supplied). Renaturing Buffer was replaced with 100 mL 1x zymogram Development Buffer (#161-0766, 50 mM Tris, 200 mM NaCl, 5 mM CaCl_2_, 0.02% Brij-35 pH 7.5, supplied) and equilibrated for 30 min at room temperature. Development buffer was replaced with renaturing buffer and incubated overnight at 37 °C for maximum sensitivity.

Zymogram gels test for proteolytic activity when performing protein characterization. The varying gel compositions act as differing substrates for proteases separated in the gel. Gels were stained in R-250 Coomassie Brilliant Blue R-250 for 1 h and stored in 100 mL Milli Q water. Gelatinases, Matrix Metalloprotease (MMP) 2 and MMP 9 can be detected via 10% Zymography, while MMP 1, MMP 7, MMP 12, and MMP 13 are detected via 12% Zymography. By performing both Zymography tests with the identical venom concentrations under identical conditions, direct comparison between venoms can be made.

#### 4.3.3. sPLA_2_ Assay Kit Cayman Activity

Triplicate venom concentrations of 1 µg were prepared, along with the of positive control bee venom supplied with the Cayman 765001 assay kit. Triplicates were used instead of duplicates to strengthen the statistical power. Supplied methods consisted of non-enzymatic controls, positive controls and sample wells. Non-enzymatic controls were prepared using 10 µL DTNB (10 mM DTNB in 0.4 M Tris-HCl, pH 8.0) (supplied) and 15 µL Assay Buffer (25 mM Tris-HCl, pH 7.5, 10 mM CaCl_2_, 100 mM KCl, 0.3 mM Triton X-100). Positive control wells were prepared using 10 uL DTNB, 10 uL provided bee venom (100 µg/mL) and 5 µL Assay buffer. Sample wells consisted of adding 10 µL DTNB, 10 µL sample and 5 µL Assay Buffer. Reactions were initiated by adding 200 µL Substrate Solution (supplied) to all wells. Samples were prepared in a 96 well plate and run at A-405 nm every minute for 10 min using Fluostar Optima absorbance (BMG Labtech, Ortenberg Germany). Data was analysed using MARS data analysis software (BMG Labtech, Ortenberg Germany) and results were published using PRISM software graphs.

## Figures and Tables

**Table 1:** Toxin types recovered by shotgun mass spectrometry, arboreality, and typical total body lengths of species under study. Body length information is from Campbell and Lamar (2004) [131].

Toxin types	aur	lat	mar	sch	bil	tan	asp	neu
5’ nuc				X			X	X
BPP/CNP	X		X			X		X
CRiSP	X			X			X	X
Kallikrein	X	X		X	X	X	X	X
Kunitz								X
Lectin		X			X			
LAAO		X	X	X	X	X	X	X
PLA_2_		X		X			X	
PLB			X					
PD						X	X	
SVMP/Dis	X						X	X
Usual TL (cm)	<70	<80	>80	<60	<70	<100	120–180	60–70
Max TL (cm)	101	100	96.8	97.9	123	175	250	100
Arboreal	Y	Y	Y	Y	Y	Y	N	N

aur = *Bothriechis aurifer*; lat = *Bothriechis lateralis*; mar = *Bothriechis marchi*; sch = *Bothriechis schlegelii*; bil = *Bothrops bilineata*; tan = *Bothrops taeniata*; asp = *Bothrops asper*; neu = *Bothrops neuwiedi bolivianus*.

## Supplementary Materials

The following are available online at www.mdpi.com/2072-6651/8/7/210/s1.
